# Opportunities and Challenges of Promoting Scientific Dialog throughout Execution of Future Science-Driven Extravehicular Activity

**DOI:** 10.1089/ast.2018.1901

**Published:** 2019-03-06

**Authors:** Shannon E. Kobs Nawotniak, Matthew J. Miller, Adam H. Stevens, Jessica J. Marquez, Samuel J. Payler, Allyson L. Brady, Scott S. Hughes, Christopher W. Haberle, Alexander Sehlke, Kara H. Beaton, Steven P. Chappell, Richard C. Elphic, Darlene S.S. Lim

**Affiliations:** ^1^ Department of Geosciences, Idaho State University, Pocatello, Idaho, USA.; ^2^ Jacobs/NASA Johnson Space Center, Houston, Texas, USA.; ^3^ UK Centre for Astrobiology, School of Physics and Astronomy, University of Edinburgh, Edinburgh, UK.; ^4^ NASA Ames Research Center, Moffett Field, California, USA.; ^5^ School of Geography and Earth Sciences, McMaster University, Hamilton, Canada.; ^6^ Mars Space Flight Facility, School of Earth and Space Exploration, Arizona State University, Tempe, Arizona, USA.; ^7^ Biomedical Research and Environmental Science Division (SK), NASA Johnson Space Center, Houston, USA.; ^8^ Bay Area Research Institute (BAERI), Moffett Field, California, USA.

**Keywords:** Mars, Spaceflight, Science communication, BASALT, Analog

## Abstract

Science-driven, human spaceflight missions of the future will rely on regular and interactive communication between Earth- and space-based teams during activity in which astronauts work directly on Mars or other planetary surfaces (extravehicular activity, EVA). The Biologic Analog Science Associated with Lava Terrains (BASALT) project conducted simulated human missions to Mars, complete with realistic one-way light time (OWLT) communication latency. We discuss the modes of communication used by the Mars- and Earth-based teams, including text, audio, video, and still imagery. Real-time communication between astronauts in the field (extravehicular, EV) and astronauts in a communication relay station (intravehicular, IV) was broadcast over OWLT, providing important contextual information to the Science Backroom Team (SBT) in Mission Control. Collaborative communication between the Earth- and Mars-based teams, however, requires active communication across latency via the Mission Log. We provide descriptive statistics of text communication between IV and SBT in a high-fidelity, scientifically driven analog for human space exploration. Over an EVA, the SBT sent an average of ∼23 text messages containing recommendations, requests, and answers to questions, while the science-focused IV crew member (IV2) sent an average of ∼38 text messages. Though patterns varied, communication between the IV and SBT teams tended to be highest during ∼50–150 min into the EVA, corresponding to the candidate sample search and presampling instrument survey phases, and then decreased dramatically after minute ∼200 during the sample collection phase. Generally, the IV2 and SBT used ∼4.6 min to craft a reply to a direct question or comment, regardless of message length or OWLT, offering a valuable glimpse into actual time-to-reply. We discuss IV2-SBT communication within the context of case examples from an EVA during which communication failures affected operations in the field. Finally, we offer recommendations for communication practices for use in future analogs and, perhaps, science-driven human spaceflight.

## 1. Introduction

Successful human spaceflight missions have depended on effective communication between teams of Earth-bound support personnel and in-space astronauts. On the other hand, Mars robotic exploration is successful with significantly less communication, using limited, intermittent bandwidth and experiencing communication latencies in the order of several minutes. In exploring how the different communication cadences might affect future human spaceflight missions that are scientifically focused, planetary analogs have provided one avenue to better understand the critical dependencies that exist between a Science Backroom Team (SBT) in Mission Control and the astronaut crew (Eppler *et al.,* 2013; Chappell *et al.,* 2016; Miller *et al.,* 2016). Science-focused missions will include a host of specific scientific priorities across a variety of scientific disciplines, guided by a multitude of scientific experts who will remain Earth-bound while astronauts attempt to satisfy those objectives. How might scientific expertise that resides in the Earth-based Science Backroom Team (SBT) influence astronauts' extravehicular activity (EVA) execution and vice versa? In particular, we consider the use of intra-EVA communication such that SBT are able to influence activities on Mars in near real time, rather than limiting to the inter-activity cadence more typical of modern robotic exploration.

Complicating the ability to communicate between SBT and astronauts located at future deep-space destinations is the communication latency between them. Due to the orbital paths of Earth and Mars, one-way light time (OWLT) ranges from approximately 4 to 22 min between the two planets. The NASA BASALT (Biologic Analog Science Associated with Lava Terrains) research program studied two latency conditions, 5 and 15 min OWLT as detailed in the works of Lim *et al.* (2019) and Beaton *et al.* (2019). Prior NASA analog research tests have identified a variety of complications associated with time-delayed communication including confusion of sequence of tasks, interruptions, wasted time waiting for responses, and an overall impaired ability to provide relevant information (Love and Reagan, 2013). Thus, a question asked by the team on “Mars” might not receive an answer from a team on Earth for 30 min, leaving little leeway for requests for clarification or identification of misunderstandings under time-pressured situations. In this paper, we offer an analysis of actual response times exhibited by personnel under both OWLT latency conditions.

**FIG. 1.**
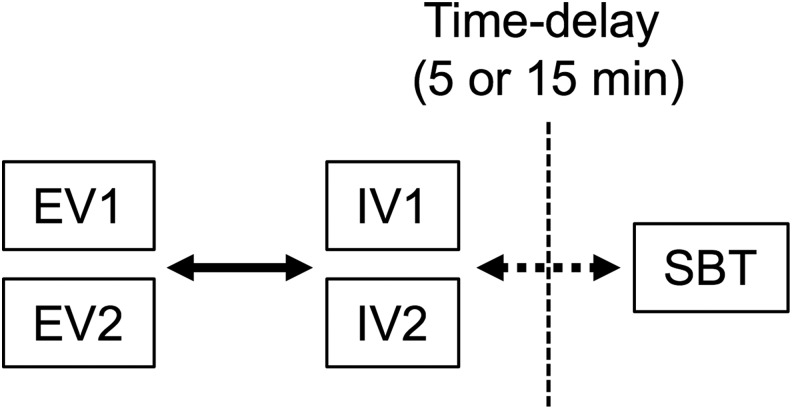
Simple schematic of the EV, IV, and SBT teams with regard to communication latency during an EVA. The EV and IV teams on the left side of the dashed line are on “Mars” and communicate with each other without OWLT (solid arrows). The SBT is located on “Earth” and communicates with the IV team through latency (dashed arrows).

This paper explores the communication patterns, modes, and frequency of Earth-Mars communication exhibited during the EVA simulations conducted by the BASALT project, with an emphasis on lessons learned from communication failures and mitigation strategies employed to avert the challenges associated with communication latency. In particular, we focus on the communication between SBT and the science-focused intravehicular astronaut (IV2) who served as a communication relay to extravehicular astronauts (EV) from the relative safety of a local Mars habitat, following a concept of operations design explained in detail in Lim *et al.* (2019) and Beaton *et al.* (2019) (Fig. 1). We provide a statistical analysis of text-based scientific communication in a high-fidelity exploration analog, offering insight into application in future latency-affected human spaceflight missions.

### 1.1. Communication structure in BASALT simulations

Communication during a BASALT EVA simulation followed a prescribed structure where SBT and IV2 could send and receive text messages (in addition to annotated imagery) across predetermined communication latencies consistent with Earth-Mars distances (*e.g.,* 5 or 15 min OWLT, relative to ∼4–22 min OWLT depending on orbital position) (Beaton *et al.,* 2019; Lim *et al.,* 2019). The task of text messaging in SBT was assigned to the Science Communicator (SciCom), though often it was the Science Lead who composed the messages intended to convey scientific inputs and/or rationale from the SBT. The messages were proofread for clarity and grammar by the SciCom prior to being sent to IV. On Mars, IV2, the astronaut scientist, served as the focal point for SBT message processing while also maintaining audio communication with IV1 (operations astronaut) and the EV crew. All verbal exchange between the IV/EV crew, synced to the associated OWLT-delayed video, was streamed to the SBT, who listened passively for supplemental operational and scientific details. While IV-EV teams trained together and practiced the communication protocols, BASALT EVA simulations were not controlled for team variability, training experience, or background. For a more detailed description of the communication modes and study conditions, refer to Beaton *et al.* (2019).

### 1.2. Phases of an EVA

Each EVA timeline was decomposed into a series of timed phases defined in terms of EV actions. These phases ensure that the SBT has sufficient opportunity to ingest and evaluate information before deciding on recommendations. A brief summary of the phases is provided here for context; for a thorough explanation, please refer to the work of Beaton *et al.* (2019). The EVA began with (1) translation to the intended study sites, called stations, which were defined prior to the deployment using precursor data (Beaton *et al.,* 2019). This phase provides opportunity for EV to describe the larger context of their surroundings, as well as identify potential targets of opportunity, thereby improving the situation awareness of the IV and SBT. Upon arriving at the edge of the intended station, EV crew (2) provided a detailed contextual description without entering the site and risking sample contamination. EV crew then proceeded into the station to (3) search for and propose candidate sample locations. This included verbal descriptions, photographic documentation, thermal measurements, and markers placed at proposed sample locations. The candidate sample location search was repeated at up to two additional stations, depending on station size. By the completion of the candidate sample search, SBT had sufficient time to down-select proposed sample locations from the first two stations and request specific targets for the next phase, (4) a presampling survey of proposed locations using handheld spectrometers (Sehlke *et al.,* 2019). SBT used the incoming mineralogy and geochemistry data to determine which of the proposed samples they wanted to recommend for actual collection. In the final phase of the EVA, (5) EV crew used aseptic techniques to collect the samples.

## 2. Modes of Communication and Perceived Value

Several communication modes were used to convey information and data between Mars- and Earth-based crew: GPS tracking, voice, video, stills (some with annotations), and text. The application and perceived value of each mode are discussed below.

### 2.1. GPS tracking

The two EV crew each carried a GPS antenna in their gear that allowed continuous tracking of their position and trackline display on aerial imagery (∼20 cm/pixel) available from Bing or Google Earth. Like all other modes of communication, position tracking was transmitted to the SBT across the OWLT communication latency. The GPS provided 1.5 m horizontal resolution (Miller *et al.,* 2019); however, spatially variable base image registration introduced an additional ∼1–3 m apparent error that became most apparent when an EV crew appeared to traverse across a cliff edge. Thus, the GPS tracking aided the IV and SBT crew in understanding the general position of EV in the field.

### 2.2. Voice communication

Voice communication during the BASALT EVAs included two distinct forms: passive listening to transmission of the EV-IV audio/video feed by the SBT and direct voice messages sent between IV and SBT which were spoken through the communication system, ensuring that messages were delayed appropriately for the EVA's OWLT latency conditions. While the former was a near-continuous presence throughout an EVA, use of the latter was discontinued by the BASALT team during the first deployment due to issues described below.

**FIG. 2.**
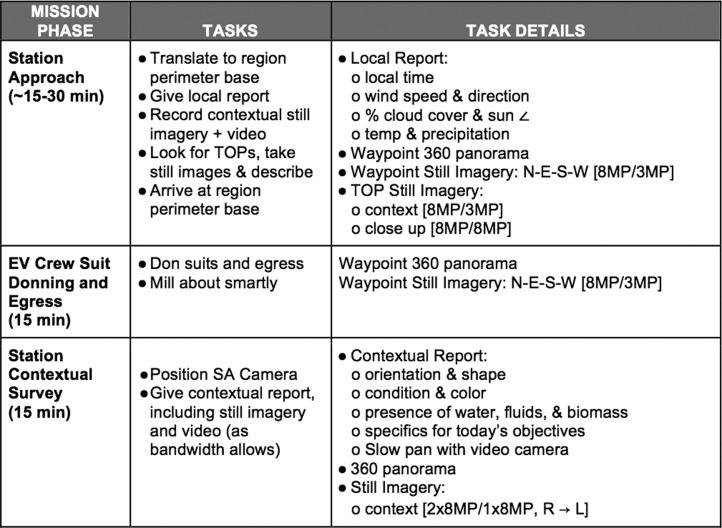
Example of a cue card carried by the EV team during an EVA. Content was divided by mission phase and reminded crew of the tasks associated with each phase, including observations and measurements to verbally state to IV over their shared live-time communication feed.

The content of communication on the EV-IV channel included EV descriptions of the field area, preferences for sampling, and updates on the condition of the EV team. Figure 2 is an example of a cue card carried by the EV team to remind them of specific tasks and observations verbalized on the EV-IV channel. A more detailed discussion of observations/data transmitted from the field and how they were used in tactical decision-making is covered in the work of Stevens *et al.* (2019).

Transmission of direct voice messages between SBT and IV, tested and abandoned early in the Idaho 2016 deployment, proved unpopular with IV due to the intrusive nature of the design; it was not reintroduced in Hawai‘i 2016 or 2017. When tested in Idaho, IV would receive an audio transmission prompt from SBT announcing an incoming message would arrive in 30 s. This allowed the IV personnel to prepare for the incoming message. Audio messages were not prerecorded but were sent via latency delay. As a result, there was no opportunity for playback if the message was not heard properly the first time. IV team members reported significant workflow disruption in order to listen and reply over the SBT-IV audio channel, and were dissatisfied that messages had no permanence for later review. All voice messages of this kind were initiated by SBT, and the replies from IV amounted to acknowledgement of receipt. Thus, SBT did not experience the same degree of disruption from using this mode of communication. While the use of a voicemail style system, rather than continuous feed, could improve this communication mode, limitations in content preview and searchability would likely remain. As such, text messaging became the primary means of communication rather than developing improvements to audio transmission. The BASALT team rated voice communication as unacceptable over latency delay (Beaton *et al.,* 2017). This is consistent with findings from past analog research (Love and Reagan, 2013; Rader *et al.,* 2013), which have also found that time-delayed audio communication across significant latency is suboptimal.

### 2.3. Video transmission

For the BASALT research program, one-way video transmission from EV crew to IV, and subsequently SBT, was implemented. The EV crew wore chest-mounted video cameras that provided a constant feed to IV. During high-bandwidth conditions, the video feed was transmitted to SBT over appropriate latency delay; for low-bandwidth conditions, SBT did not receive video. The video cameras were not capable of pan/tilt/zoom beyond that provided by the movement of the EV crew. As such, the video largely existed as a passive feed that offered an approximately first-person view from the EV perspective. During EVA tasks that only required one EV crew member, such as breaking a rock with a hammer, the other EV would stand back and use their video feed to provide a view of what their partner was doing.

In addition, the EV team was followed by a tripod-based video camera that provided a contextual view analogous to one that might be provided by the mast camera on a rover (see Lim *et al.,* 2019; Miller *et al.,* 2019). Like the EV chest-mounted cameras, the tripod-based camera provided continuous transmission to IV and bandwidth-dependent transmission to SBT. The tripod-based camera was carried by a member of the support personnel who could receive direction on camera placement and orientation from EV or IV but was otherwise unable to communicate within the simulation.

While the video feed was a predominantly passive mode of communicating information from the field to IV and SBT, it was sometimes used by EV in more active forms. For instance, EV crew would intentionally use their video cameras to provide close views of their partner during sample collection, allowing IV and SBT to monitor for possible violations of sterile collection protocol. EV would often use their video feed to enable discussion with IV2, for example asking for feedback on their interpretation of a specific rock sample. During an unexpected failure of the still imagery camera in the field, EV and IV compensated for the loss of still imagery by holding stationary and aiming the video cameras at targets of interest in order to facilitate frame captures directly from the video feed.

IV2 reported extensive use of the video feeds during EVA in order to understand the actions and context of the EV team, as well as to make scientific interpretations. Conversely, however, many members of the SBT reported only minimal use of the video feed when it was available. As such, the video feed was primarily useful during the BASALT EVAs for EV-IV communication and served a lesser role for SBT during their tactical decision-making (see Stevens *et al.,* 2019). The limited use of the video feed by SBT during EVAs may have been at least partially a result of the limited scientific detail found in the video feed as compared to the high-resolution still imagery. As a result, SBT attention and resources were applied to more descriptive data sources such as imagery and instrument measurements.

### 2.4. Text messaging

The scientific discussion between SBT and IV2 occurred in the form of text messages via the Playbook Mission Log software, similar to prior analog simulation including Pavilion Lake Research Project (PLRP) and NASA Extreme Environment Mission Operations (NEEMO) (Marquez *et al.,* 2013, 2019). The Mission Log enabled users to send and receive text messages while automatically accounting for the communication delay, displaying messages arranged in a vertical timeline ordered by “sent time stamp” that displayed local 24 h format time to the second and was synced across all platforms. In addition to labels indicating who sent the message and at what time, the Mission Log also displayed countdown clocks on sent messages to indicate when the recipient would actually receive the message and what time to expect the earliest possible response. For the Hawai‘i deployments, updates to Playbook allowed the team to send messages as either regular or high priority; high-priority messages were displayed in bold font and persisted at the top of the Playbook Mission Log screen until dismissed by the recipient. Optional message headers (Table 1) were also used, particularly by SBT, in order to clearly indicate the intent of each SBT message; this was particularly helpful to distinguish quickly between messages indicating SBT priorities for presampling survey versus sampling phases, which otherwise looked similar in content and structure. The headers were predetermined before the mission and were copied out of a text file of standardized options or typed free-form by the sender. The message headers improved content searchability, allowing IV2 and SBT to more quickly navigate communication records, and could be used to create filtered lists of all messages with the same header. The ability to filter was particularly useful in cases where multiple iterations of the same type were provided, for example, priority updates from SBT.

**Table 1.** Message Headers that SBT Used to Flag Text in the Playbook Mission Log

*ST MESSAGE HEADERS*	
^***^ SCIENCE SURVEY PRIORITY ^***^	^***^ SCIENCE REQUEST ^***^
^***^ SCIENCE SAMPLING PRIORITY ^***^	^***^ SCIENCE NOTE ^***^
^***^ SCIENCE PRIORITY JUSTIFICATION ^***^	^***^ SCIENCE CAUTION ^***^
^***^ ST PRE-SAMPLING PRIORITY ^***^	^***^ SCIENCE WARNING ^***^
^***^ ST PRE-SAMPLING PRIORITY JUSTIFICATION ^***^	^***^ SCIENCE RESPONSE ^***^
^***^ SCIENCE REMINDER ^***^	^***^ SCIENCE INQUIRY ^***^
^***^ SCIENCE RECOMMENDATION ^***^	

Bold font indicates that the message was sent by default as high priority (bold text in message, highlighted message box).

**FIG. 3.**
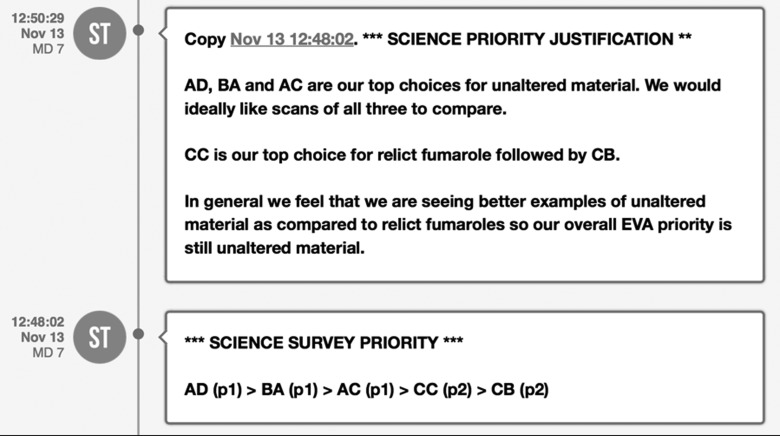
Examples of messages from SBT (labeled as ST in the Mission Log) to IV2 indicating priority ranking of candidate sample locations for closer examination in the presampling instrument survey, followed by a justification of the priority order. Letters AD, BA, AC, CC, and CB refer to sample location marker cards placed in the field by EV. The tags in parentheses after each location name indicate whether that candidate sample is under consideration for science priority 1 or 2 for that EVA; in this example, the priorities are (1) unaltered lava and (2) relict fumarole. In these messages, SBT decided to emphasize the importance of the justification text by using the high-priority flag to make the text bold and place a blue highlight on the message box.

**FIG. 4.**
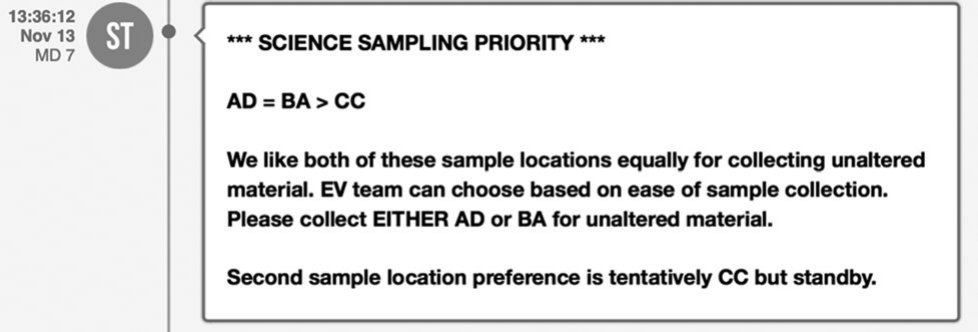
Example of a combined priority ranking and justification. This message occurred later in the same EVA as the messages in Fig. 3 and includes a shorter list of sample priorities as SBT down-selected to the top candidates. AD and BA are given equal consideration by SBT for unaltered sample collection, allowing EV to make a final decision in the field about which to sample. The final line gives temporal context that SBT has not yet evaluated all presampling survey instrument data that could influence the decision between CC and CB.

Text message communication between IV2 and SBT was a convenient solution to the challenges of communicating over latency because they could be read and reread at a time of the reader's choosing. Time stamps attached to each message enabled the reader to associate when in the message log the message was actually sent (Marquez *et al.,* 2019). The most commonly reported challenge regarding text messaging concerned the difficulty of rapidly and accurately typing messages while managing multiple other interactive channels of communication, for instance IV2 simultaneously interacting over the audio and video feeds with EV while messaging with the SBT. Time pressure to send messages quickly sometimes resulted in unintended typos or word choices that were inadvertently vague or misleading. During the BASALT deployments, attempts were made to mitigate this problem by having a second person, the SciCom, review text messages before they were transmitted out of SBT; IV2 lacked the resources to institute the same policy (Marquez *et al.,* 2019). Additionally, mutually acceptable shorthand communication for specific periods through the EVA timeline was developed to convey the necessary content at specific times during execution, such as sample priority decisions made during the candidate sample selection phase (Figs. 3 and 4).

### 2.5. Still imagery

**FIG. 5.**
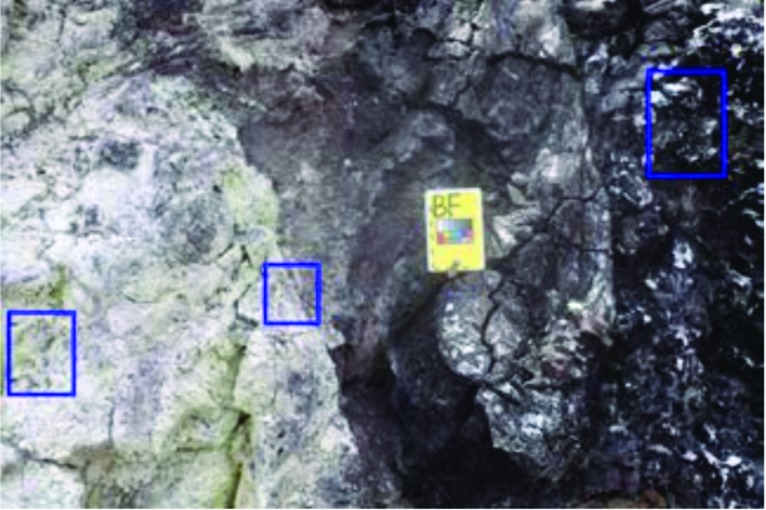
An example of an annotated photo sent through the Playbook Mission Log by SBT to indicate specific sampling preferences. In this case, SBT was requesting a geology (nonsterile collection) sample from each of the areas indicated by the blue boxes, spanning an alteration gradient in the basalt. Explanatory text was sent as regular Mission Log messages. Note that the blurriness of the image is consistent with how the image appeared when it was opened in the Mission Log during the EVA.

Still imagery from the field was highly valued by SBT, regardless of whether the study condition allowed EV video transmission to Earth. Still images, taken at 3 and 8 megapixels, provided various scales of context (landscape, outcrop, or close-ups of specific features; “point-like” and “linear” topologies of Kereszturi, 2011) and provided SBT with the opportunity to conduct their own detailed observations of proposed sample locations. On rare occasions, annotated still images were sent through the Playbook Mission Log by IV2 or SBT in order to direct attention to specific features in the photographs taken by EV (Fig. 5). While the team deemed it beneficial to send annotated images between SBT and IV, the available software tools were not purpose built to facilitate this task directly. Instead, multiple commercial software packages were used to generate and exchange annotated images to be sent in the Mission Log; specific software packages for image annotation were not tracked, as they varied by what individual users already had available on their laptops, but included the Microsoft Office suite, Preview, and Paint. Difficulties included quickly transferring images into annotation-capable software, uploading annotated imagery into the Mission Log (for SBT, this meant transferring files to the Science Lead or SciCom personnel), and producing clear annotations if the user tried to do something more detailed than inserting a box to highlight a specific feature. Annotations were kept simple, with supplemental explanatory text written in the Mission Log entry associated with the image. This ensured that explanatory text was connected to the image in the Mission Log, without potentially decreasing legibility by placing text directly onto the image.

## 3. Communication Behaviors Associated with Text Messages

### 3.1. Messages from SBT to IV2

Text messages originating from SBT consisted of a variety of content that ranged from specific requests, suggestions, or directions intended for IV2 to pass to the EV crew. For example, SBT used text messages to send priority update lists for both presampling survey instrument scans in the field and final sample selection. Messages were fairly concise and encapsulated the consensus reached by the team on Earth (*e.g.,* Figs. 3 and 4; Payler *et al.,* 2018).

Each time new information about a candidate sample location was assessed and integrated into the SBT priority list, an updated priority list was sent to IV2. These periodic updates were intended to (1) provide IV2 insight into the decision-making rationale SBT used within the mission objectives for that EVA or (2) convey a clear reason for deviating from the intended objectives, such as in situations when the targeted sample type did not appear to be present. The SBT also noted the strength of their decision by indicating strong or weak preferences by explicitly stating comments that conveyed such sentiments (see Fig. 4 for an example in which SBT indicated no preference between two candidate sample locations and left the final decision to EV/IV).

Coupled with message conviction, message intent was denoted by headers such as “Science Request,” “Science Recommendation,” and “Science Note,” with the highest-priority messages given bold font headers (Table 1). In practice, however, many of these headers were used inconsistently or not at all; in particular, SBT tended to skip headers on low-priority messages or ones that were in direct reply to IV2 messages due to the limited perceived benefits. Headers were most helpful in messages that contained novel, important content. It was also helpful for SBT messages to indicate when in the timeline they were sent from, whether explicitly or via context. In doing so, the reader could parse out what was and was not known by the sender to better situate if those assumptions were still valid.

### 3.2. Messages from IV2 to SBT

While SBT overheard all the verbal communication between EV and IV crew members, the need for discussion, synthesis, and decision-making in SBT during the continuous audio feed from Mars created distractions and opportunities for missed communication (Payler *et al.,* 2019). While this would be mitigated to some extent in actual flight operations via extensive SBT console training (*e.g.,* learning how to manage multiple audio conversations at once), IV2 was responsible for distilling key pieces of scientific information for SBT via the Playbook Mission Log to ensure pertinent information about ongoing operations was not missed. This content included transcribing output read aloud from field instruments that did not transmit directly into Minerva, the data management system used in BASALT (Marquez *et al.,* 2019), writing short descriptions of the individual candidate sample locations, and copying sample priority preferences stated by EV. During low-bandwidth conditions, in the absence of video transmission, IV2 also provided their own insights into the geological context of the area or any features of interest that appeared in the video.

In addition to sending messages to SBT, IV2 compared incoming SBT messages against their own notes (which included their own sampling priority order that they anticipated based on field observations relative to EVA science objectives) to identify possible discrepancies that may indicate a misunderstanding between crews on Earth and Mars. Based on these comparisons, IV2 pointed out potential problems to SBT or requested clarification as needed. There were some instances where the SBT priority order differed from IV2 expectations as a result of the multidisciplinary scientific discussion in the SBT during the EVA; in other instances, IV2 successfully identified communication discrepancies. These situations were resolved by sending explicit questions or clarification messages in the Mission Log under communication latency, potentially resulting in lost time from an EVA. In the event that the EV/IV teams did not believe that there was sufficient time to allow for a response from SBT, they were allowed to make an independent decision rejecting the possibly incorrect guidance from SBT or to modify task priorities in order to create additional time for a question and reply; while the latter happened on a couple of occasions, the “Mars” teams did not choose to use the former option and override SBT guidance, even when they believed it to be incorrect.

### 3.3. Aggregate summary statistics of text communication behaviors

**FIG. 6.**
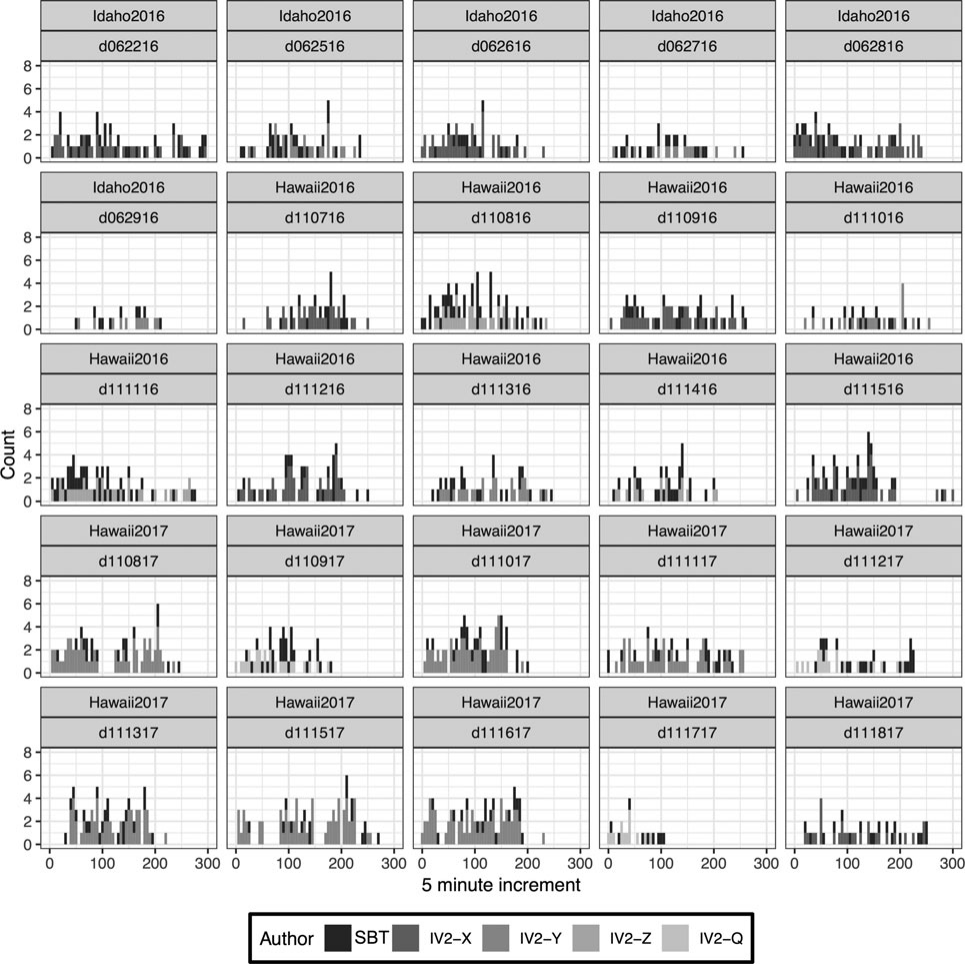
Histogram of communication instances divided by EVA across all three deployments (Idaho 2016, Hawai‘i 2016, Hawai‘i 2017) binned into 10 min increments. SBT = Science Team; IV2-X, IV2-Y, IV2-Z, IV2-Q denote the unique crew member fulfilling their role as an intra-vehicular science member. Note that EVA d111817 followed a different EVA structure than the others.

Various communication patterns between SBT and IV2 were exhibited across each EVA performed during BASALT. These communication statistics are descriptive and not part of a controlled experiment or analysis. The intent is to characterize communication patterns and not to conclude requirements or effects (unlike Smith *et al.,* 2008). Figure 6 shows the frequency count of messages binned into 10 min increments throughout each EVA. EVAs d062016, d062116, d062316, and d062416 did not meet the simulation quality criteria specified in the work of Beaton *et al.* (2019), therefore were omitted from the following analyses. Additionally, the final EVA, d111817, followed a unique timeline due to testing with virtual and augmented reality capabilities. Four different scientists (X, Y, Z, Q) served as IV2 across these deployments. SBT messages were crafted and sent by the Science Lead or SciCom (see Payler *et al.,* 2019), representing the consensus of the SBT; due to the group representation and number of personnel who engaged in the Science Lead and SciCom roles, SBT message statistics are reported here as a single group rather than broken down by personnel like for IV2.

The communication cadence between SBT and IV2 exhibited a variety of patterns throughout what was otherwise a consistent timeline structure used across the EVAs (Fig. 6). Periods of high and low frequency of communication shifted based on unique conditions such as distance covered, weather, and ease of sample identification and collection during each EVA. As a result, the communication demands varied on a per-EVA basis even though the same operational timeline was used for each EVA. Aggregate communication characteristics are shown in Table 2.

**Table 2.** Communication Instance Summary Statistics across the BASALT Program

*Mission*	*Author*	*# of EVAs*	*Total # of messages*	*Messages per EVA*	*Avg. word count*	*95% CI word count*
Idaho 2016	SBT	6	126	21.0	33.1	4.2
	IV2-X	3	129	43.0	24.2	2.9
	IV2-Y	3	53	17.7	15.7	3.5
Hawai‘i 2016	SBT	9	239	26.6	23.4	2.6
	IV2-X	4	162	40.5	23.5	3.6
	IV2-Y	2	49	24.5	18.0	4.3
	IV2-Z	3	86	28.7	26.3	4.2
Hawai‘i 2017	SBT	10	199	19.9	27.3	2.7
	IV2-X	1	27	27.0	33.1	8.3
	IV2-Y	6	378	63.0	18.7	1.2
	IV2-Q	3	58	19.3	36.6	6.0

Over the three deployments, IV2-X and IV2-Y, the most frequent people in the role, averaged nearly the same number of messages per EVA. However, the rate of messages from IV2-X started high and decreased over time, while the reverse was true of IV2-Y. IV2-Z and IV2-Q, who had the fewest rotations into the position, averaged fewer sent messages per EVA but typically used more words per message than IV2-X and IV2-Y. Some of these differences can be attributed to evolving Mission Log capability, with the introduction of an “Acknowledge” feature that reduced the need to send messages indicating receipt in the Hawai‘i 2017 deployment.

**FIG. 7.**
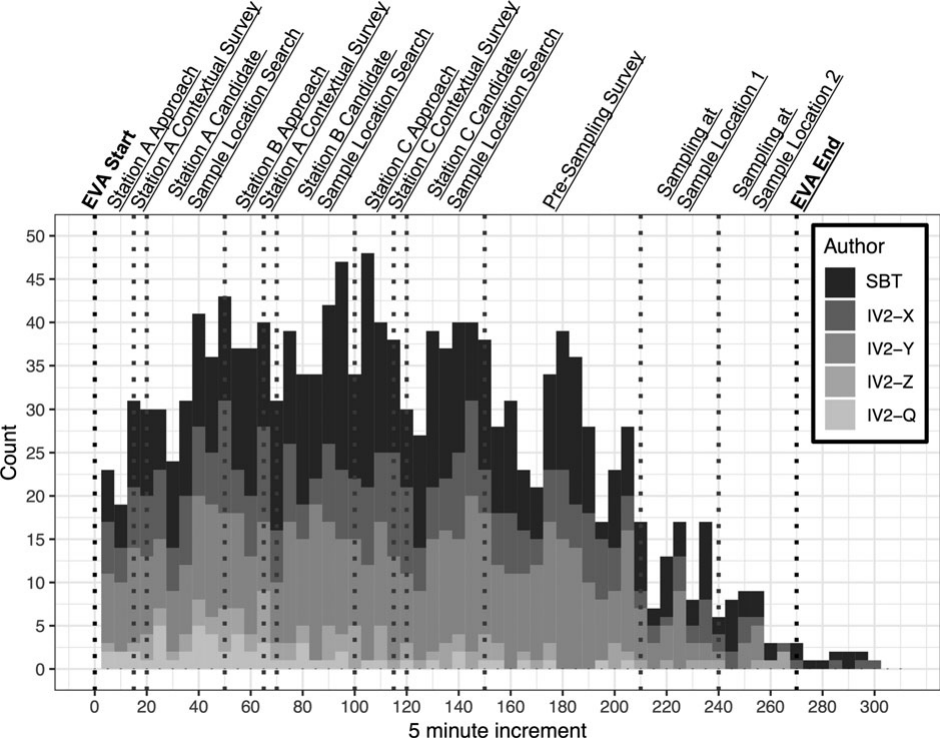
Aggregated histogram of communication instances across all the entire BASALT program binned into 10 min increments. SBT = Science Team; IV2-X, IV2-Y, IV2-Z, IV2-Q denote the unique crew member fulfilling their role as an intra-vehicular science member.

In terms of aggregate behavior (Fig. 7), all authors exhibited their highest period of communication exchange between ∼50 and 150 min into the EVA, which maps to the candidate sample search and presampling instrument survey phases. At approximately 200 min, there is a distinct diminishing of text communication. This is attributed to the BASALT EVA timeline design: once the EV crew entered the sampling phase, there was little opportunity for SBT to influence the EVA due to latency limitations. As such, SBT was significantly less likely to initiate messages. Similarly, IV2 was occupied logging samples into the Minerva software system during the sampling phase and less likely to send Mission Log messages to SBT.

**FIG. 8.**
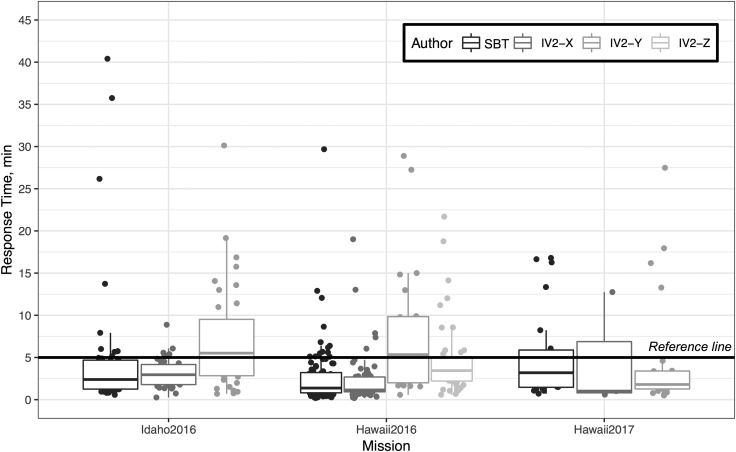
Box plot of response time for each response message sent by the SBT and IV2. The minimum word count for a copy message was 6, which was the standard copy with time stamp message automatically generated by the Mission Log.

The ability to respond to specific Mission Log messages during an EVA is paramount to ensuring all inquiries or requests are satisfied in a timely manner. During the deployments, the BASALT team was instructed to use the “Copy” feature within the Mission Log to indicate receipt and comprehension of, or to reply directly to, a specific text message; while the entire team was trained to use “Copy,” not all team members complied. The Copy feature in the Mission Log generated a reply text that included the word “Copy” and the time stamp of the original message, which also served as a hyperlink back to the original (Marquez *et al.,* 2019); the user could add additional text as desired. Figure 8 shows the distribution of each copy message as a box plot; Table 3 shows summary statistics by sender across the three deployments. Note that all time-delay conditions have been removed from the data such that there is no bias for different latency conditions used in simulations; therefore, the response time axis (Fig. 8) is indicative of the total “working” time required for an author to notice, open, read, interpret, and generate a response to a received message, starting from the moment of arrival in their Mission Log. The response time data is included here to establish a realistic lower limit on what can be expected of (1) a science team to arrive at consensus and supply a concise response and (2) an IV operator to respond to messages while also performing a multitude of other duties. Outliers in Fig. 8 may have originated from failing to notice an incoming message, forgetting to use the “Copy” feature until later in an EVA, task overloading during a period of heavy activity, or some other reason; identification of the cause of each outlier is beyond the scope of our analysis and, in many cases, would only have been identifiable by close observation at the time of the incident.

**Table 3.** Aggregate Copy Message Response Time and Word Count Statistics for Each Author across All BASALT Deployments

*Mission*	*Author*	*# of messages*	*Average response time (min)*	*95% CI*	*Word count*	*95% CI*
Idaho 2016	IV2-X	37	3.1	0.6	23.7	4.7
	IV2-Y	36	7.4	2.2	13.7	3.9
	SBT	47	4.9	2.5	25.3	5.7
Hawai‘i 2016	IV2-X	64	2.2	0.8	12.2	3.2
	IV2-Y	23	7.7	3.5	8.2	2.7
	IV2-Z	43	4.8	1.5	21.3	3.8
	SBT	92	2.7	0.8	13.7	3.3
Hawai‘i 2017	IV2-X	3	4.8	17.7	10.3	15.1
	IV2-Y	24	4.7	3.0	15.7	3.0
	SBT	22	5.3	2.5	26.7	4.8

Note that IV2-Q is not included in the table, as they served in the role after the implementation of the Acknowledge feature and did not utilize the Copy feature.

When considering response times for both 5 and 15 min latency conditions, SBT on average replied to messages in 4.4 (6.8 s.d., *N* = 96) and 2.6 (5.1 s.d., *N* = 65) minutes while the IV2 on average replied in 4.4 (5.9 s.d., *N* = 135) and 4.6 (4.7 s.d., *N* = 95) minutes, respectively. This explanatory data provides one valuable avenue to unobtrusively measure the capabilities of operators while working on these simulated EVA conditions that could serve as a future benchmark to promote the timely delivery of responses to messages.

Across each deployment, the BASALT program exhibited a reduction in the total count of copy messages (Table 3). Further, an “Acknowledge” feature was added to the Mission Log prior to the Hawai‘i 2017 deployment; this feature replaced the use of “Copy” messages that did not include additional text, such as those simply acknowledging receipt of a message. The inclusion of “Acknowledge” reduced the total number of “Copy” messages that would have otherwise been sent.

## 4. Scientific Communication Discrepancies Experienced during EVA

Communication discrepancy events from one EVA, as described below, serve as an illustration of the various dynamics that can take place during an EVA. In these specific instances, the IV2 was in a unique position relative to the rest of the team to identify and resolve miscommunication that could result in scientific impedance during the EVA. The intent of this section is not to provide a comprehensive description of discrepancies or solutions but rather initiate the discussion surrounding what future EVA operations might encounter as scientific objectives become the focal point of discussion. These case-study events serve as a basis for what will surely be an ongoing and worthwhile research endeavor into promoting scientific dialog in future missions.

### 4.1. Candidate sample location placement

On mission day 8 of the 2016 Hawai‘i deployment, the EVA objectives were to collect a full sample suite from (1) an active fumarole, (2) syn-emplacement alteration caused by gas exsolution and escape from the lava, and (3) if priority 2 was not available, either collect a sample suite a few meters from the priority 1 samples to capture the gradient of alteration, or repeat priority 1 at a second fumarole, depending on availability in the field. The EVA was conducted under 5 min latency and high-bandwidth conditions, meaning that EV video was transmitting to SBT on Earth.

#### 4.1.1. Confusion from SBT guidance

During the contextual survey phase, SBT noticed a bright red patch of syn-emplacement alteration in the video feed and in the north-facing contextual still image. The rock in question was previously identified as a target of opportunity (TOP) during the EVA the day prior but not sampled at that time. SBT sent a message to IV2 noting that they could see the old TOP in the image and requested that EV place a sample candidate marker at that spot if they had not already done so. The message was sent as high-priority text to ensure that IV2 addressed this request.

Because of the time latency, EV had already placed the first location marker (CA) for that station at the old TOP. SBT saw this occur in the video approximately 1 min following transmission of their request, or 4 min prior to IV2 receiving the message from SBT. Due to the quick succession of events, IV2 lacked the temporal context to know whether the SBT message had been sent before or after marker CA had been placed, thereby causing them to think that the SBT request referred to a different patch of discoloration in the context photos. IV2 directed EV to further search the area near the perceived location and to ask for clarification from SBT by sending an annotated photo. Instead of waiting the full 10 min required under low latency for a reply from SBT, IV2 only allowed EV a few minutes for the additional search before directing them back to the original tasks. Thus, IV2 created an opportunity for EV to quickly satisfy the perceived request from SBT if possible, without allowing the failure in communication to significantly delay the EVA.

This particular discontinuity in communication could potentially have been prevented entirely had the pre-EVA mission brief (Brady *et al.,* 2019) included a review of points of interest observed during the previous EVA. Otherwise, SBT should have trusted the EV team would identify the obvious patch of altered rock in the station and only submitted their request when it was clear that EV had not done so. The enthusiasm of SBT and their desire to directly control the situation on Mars resulted in confusion for EV and IV, costing a few minutes of simulation time. Furthermore, SBT could have taken the time to create and send an annotated photo in order to remove all ambiguity. However, the process to annotate photos was time-consuming, and the SBT was not inclined under such time-pressured circumstances to spend extra resources on what was perceived as a fairly obvious alteration TOP.

#### 4.1.2. SBT misled by video

Immediately following the previous incident, there was a miscommunication driven by the audio and video feeds. The EV team, returning to the regular candidate sample search, discussed whether or not to place marker CB at a specific position. EV discussed the merits of the location, including a full location description typical of a proposed sample location, while holding the CB marker. EV eventually decided that the position of the rock in question (under an overhang) was potentially hazardous for sampling and did not place the marker at that location.

SBT, having heard a full sample location description and seen both the candidate location marker and altered rock in the video feed, believed that EV had placed marker CB; no one in SBT heard the EV decision to not place the marker after all. Based on the description and video imagery, and unaware of the potential sampling hazard at that location, SBT identified the discussed CB sample as an ideal sampling target. Additionally, due to intermittent upload delays, the SBT chose not to wait for still image confirmation before ranking CB as the highest priority for instrument scans during the presampling survey. Thus, they prematurely sent the updated priority list to IV2 as well as a Mission Log message indicating their particular enthusiasm for that candidate sample.

EV, meanwhile, continued their candidate sample search and placed the CB marker elsewhere. As the audio and video feeds from the actual placement of marker CB reached Earth, SBT was thrown into confusion about the status of CB. SBT sent a high-priority science-warning message, “Do not let EV move sample markers to new positions after they have been placed/described. We are confused about whether CB was actually placed and re-used or if there was extensive description at the original location without and [*sic*] actual tag drop.”

IV2 began to identify the problem with the SBT prioritization of CB upon receipt of the priority list, several minutes before receiving the science-warning message. While the difference in time between when the priority list was sent and when the CB marker was actually placed was too small to signal an obvious problem (<5 min), IV2 did not think that the actual CB location selected was appropriate for the level of enthusiasm expressed by SBT. IV2 sent a message to SBT advising of the possible failure in communication regarding CB a full 6 min before receiving the science-warning message from SBT.

Had IV2 been confident that the CB prioritization was based on a misunderstanding, they could have overridden the apparently erroneous priority ranking. However, IV2 decided to wait for confirmation of the error from SBT before deviating from the most recent priority list. Ultimately, IV2 redirected EV ∼3 min after receiving the science-warning message, which was ∼2 min before receiving an updated priority list from SBT with the corrected CB ranking. As a result, EV spent nearly 4 min (∼7% of the allotted phase time) conducting presampling survey scans at the new, and undesired, CB before moving on to other sample locations. This loss of time was sufficient to reduce the number of sample locations that could undergo presampling survey scans by one, thereby reducing the expected number of choices available to the SBT by ∼15–20%, depending on translation time required between proposed sample locations within an EVA.

The failure in communication surrounding the CB marker might have been mitigated or avoided several ways. First, it was against EVA protocol for EV to provide so much candidate sample location description prior to placing a marker. EV was unaware that they were holding the sample markers within the field of view of the chest cameras, reinforcing SBT's belief that the marker was being placed; following this event, procedures were updated such that EV crews held markers text side down to minimize their unintentional appearance in the video frame. On Earth, SBT should ideally have evaluated the still imagery of any candidate sample location before transmitting their priority list; in the event of imagery delays like those experienced during that EVA, SBT could have double-checked the CB candidate sample location on the tracking map in the Minerva data management system. Additionally, voice-to-text transcription of the EV/IV audio feeds and video/audio playback may be possible in future missions, in which case SBT could also review transcripts in cases of doubt.

IV2 sent only 18 messages during the EVA, well below average, while SBT rates were slightly above average at 28 messages (d11142016, Fig. 6). The discrepancy regarding CB might have been prevented if IV2 had sent more messages, though there is no strong supporting evidence for this. Regular messages from SBT helped IV2 conjecture there was a misinterpretation. SBT sent 11 separate messages on their evolving prioritization and rationales for the proposed sample locations, with 9 of them sent prior to the CB event. Thus, IV2 had a better understanding of how SBT was making their priority recommendations and expected a proposed sample location would receive a lukewarm reception but instead was met with unexpected enthusiasm (“ST very excited about CB” was entered into the Mission Log).

## 5. Discussion

In the case study example, passive forms of communication (SBT observing audio and video feeds) contributed to confusion on Earth. While this was in part a result of training, since analog projects operate without full Mission Control support, staffing, and experience, we argue that modes of communication where one group observes passively through latency pose an increased risk of introducing discrepancies compared to direct modes, such as text in the Mission Log. The live-time dialog between EV and IV crew members captured by the audio and video feeds broadcast to Earth is as much an active science- and operations-driven discussion among the crew isolated on Mars as it is a report intended for the SBT. The mixing of these two purposes yields valuable human insight during the EVA but also can create verbal clutter. When some EV teams tried to differentiate between EV1-EV2 and EV-IV conversation by lowering their speaking volume or even turning off their microphones for local communication, this loss of audio was deemed unacceptable by the rest of the team; EV was explicitly required to stay on an open microphone, with limited exceptions. These experiences suggest two possible guidelines for future BASALT and potential flight missions: (1) use of a push-to-talk audio channel specifically for high-priority verbal messages, operating simultaneously with the existing open-mic channel so that SBT members can opt to listen to whichever channel better supports their task objectives, and (2) voice-to-text transcription of the existing audio feed to create an immediately reviewable record.

In all cases of miscommunication, the situation was mitigated more quickly when the Mars-based team was given authority to interpret, and even override, messages from SBT rather than operating as robotic assets. While the natural inclination of SBT may be to control what happens in the EVA, their attempts to do so across the communication latency often appeared untenable. As a result, the BASALT team renamed the “Mission Control Center” in favor of “Mission Support Center” to adjust expectations across the team. We recommend live-time dialog as a required capability that enables science, emphasizing Mars-based team autonomy.

The IV2 crew member had a particularly pivotal role in the creation and mitigation of miscommunication, positioned as gatekeeper of the flow of information between SBT and EV. An active IV2 is capable of identifying miscommunication earlier than anyone else by comparing incoming messages against their own notes. In order for this to happen, however, IV2 must (1) have a clear understanding of the EVA objectives, (2) be aware of sequences of events throughout the EVA, and (3) have developed their own priority rankings and rationales, meaning that they must also serve as a local SBT resource for the EV crew. Further, they must receive regular updates from SBT that demonstrate how the Earth-based decision process evolves throughout the EVA. This lesson learned during the early BASALT EVAs was used to train additional IV personnel in the project. The more confident the IV2 was in their own assessments and their alignment with SBT, the more likely they were to identify problems and make tactical decisions that supported EV's progress while buying time to resolve possible discrepancies. In the case of the confusion over CB (Section 4.1.2), IV2 could have directed EV to begin the presampling survey at candidate sample location CA, which was also on the priority list. This would have potentially prevented the loss of 4 min (∼7% of the allotted time) during the EVA as SBT clarified their priority list. Some IV teams in BASALT were more comfortable with this responsibility than others, particularly in instances of possible disagreement with SBT, but each of the crews was required to make strategic and tactical decisions in support of EVA objectives and timeline limitations.

To ensure that IV2 is capable of handling these additional tasks, SBT provided regular and detailed messages that demonstrate the evolving decisions and rationales of the SBT. IV2 had neither the context nor the confidence to question suspicious decisions or optimize their tactical decision-making if they only received a single priority list for each phase of the EVA. Thus, it was preferable for SBT to submit an updated priority list and rationale following each decision that they made regarding presampling survey or sampling tasks. This behavior is visible in Fig. 7, where communication rates are high for the candidate sample location search and presampling survey phases of the EVA, approximately minutes 50 to 150.

The 2-way flow of active communication is critical to EVA success and relies on text messaging across latency. Given actual distances between Earth and Mars, the round-trip reply time is 8–44 min; this does not include the time required by the recipient to read the message and craft a reply. Our results across the three deployments suggest that the average time needed to read an incoming message and compose a Copy reply is approximately 4.1 (+/− 5.5 s.d.) minutes. While seemingly a significant amount of time relative to the one-way latency, this time provides a baseline for expected response time. Other factors such as message content, crafting, and refinement likely played a more influential role in response rates. A more detailed qualitative analysis of the content of the messages and environmental factors surrounding when those messages were generated would be required to more fully understand these factors. Nonetheless, this data provides a more realistic estimate of time-to-reply capabilities of authors, thereby guiding SBT and EV/IV in tactical decision-making during an EVA. In the CB example above, the discrepancy in communication could have resulted in up to ∼14 min (5 min OWLT plus time-to-reply) lost from the 25 min presampling survey activity. The loss was minimized to only 4 min due to the decisions made by IV2 when they identified the likely failure of communication.

## 6. Conclusions

Communication between Earth and an analog Mars site during a simulated EVA involves multiple modalities, including passive audio and video feeds, still imagery, and text messages. Timeline restrictions driven by limited life support during an EVA and communication latency make efficient, clear communication critical for mission success. While the team should aspire to perfect communication without disagreements or misunderstanding, it is important to implement protocols designed to enable identification of, and recovery from, failures in communication. Toward this end, we offer the following recommendations based on the BASALT research to inform both other analog research environments and potential future missions that employ similar concepts of operations and focus on scientific objectives:

(1) Text-based communication is far preferable to audio transmission over latency, allowing message recipients to prioritize their own tasks in the moment and maintain a written record of communication for review throughout the EVA as desired.
(2) SBT should provide regular updates containing evolving information each time a decision is made regarding priorities for the EV crew. Updates should be clear with regard to the EVA mission objectives and provide brief rationales explaining decisions.
(3) Passive communication modes to Earth appear to be more susceptible to discrepancies in communication than direct communication such as text. Thus, best practices for transmitting mission-critical information from Mars should include a form of active communication or a secondary passive mode. This may involve text sent via Mission Log, voice-to-text transcription, or some other form.
(4) In the event that IV2 or SBT refers in the Mission Log to a still image taken in the field, the message should contain a copy of that image. If possible, it should be annotated for clarity.
(5) IV crew who are scientifically focused should proactively generate anticipated priority assessments based on EVA objectives and field observations. Comparing IV interpretations to SBT input is critical for identifying discrepancies in communication.
(6) Since response time to text messages was ∼4 min plus the OWLT, SBT should avoid the temptation to try to overmanage EV across latency and should instead take advantage of the crew interpretation. The OWLT limits SBT tactical decisions to reactive responses, rather than proactive, for short-term turnarounds.

## Abbreviations Used

BASALT: Biologic Analog Science Associated with Lava Terrains
EV: extravehicular astronaut
EVA: extravehicular activity
IV: intravehicular astronaut
OWLT: one-way light time
SBT: Science Backroom Team
SciCom: Science Communicator
TOP: target of opportunity
